# Thermal Management with AlN Passivation in AlGaN/GaN HEMTs with an Air Gap Gate for Improved RF Performance: A Simulation Study

**DOI:** 10.3390/mi17010092

**Published:** 2026-01-10

**Authors:** Young-Hyun Won, Tae-Sung Kim, Jae-Hun Lee, Chae-Yun Lim, Byoung-Gue Min, Dong-Min Kang, Hyun-Seok Kim

**Affiliations:** 1 Division of Electronics and Electrical Engineering, Dongguk University-Seoul, Seoul 04620, Republic of Korea; tangent10@dgu.ac.kr (Y.-H.W.); giantstar00@dgu.ac.kr (T.-S.K.); leejae00@dongguk.edu (J.-H.L.); cylk9799@dgu.ac.kr (C.-Y.L.); 2 Electronics and Telecommunications Research Institute, Daejeon 34129, Republic of Korea; minbg@etri.re.kr (B.-G.M.); kdm1597@etri.re.kr (D.-M.K.)

**Keywords:** gallium nitride, high-electron-mobility transistor, air gap gate, aluminum nitride passivation, thermal management, RF performance

## Abstract

This study introduces an air gap gate with AlN passivation to enhance the radio frequency (RF) performance of AlGaN/GaN high-electron-mobility transistors (HEMTs) while addressing thermal challenges. The air gap gate improves RF performance by reducing gate capacitance, resulting in a 23.9% increase in cutoff frequency (35.82 GHz) and enhancing saturation drain current and maximum transconductance by 3.7% and 10.27%, respectively, compared to a 0.15 μm planar gate baseline. However, reduced heat dissipation degrades thermal performance, as reflected in higher thermal resistance and temperature gradients. Incorporating high thermal conductivity AlN passivation mitigates these drawbacks, lowering operating temperatures and improving heat distribution, while maintaining a 17.5% cutoff frequency improvement over the baseline. These results demonstrate that the air gap gate with AlN passivation provides an effective strategy for achieving reliable, high-performance AlGaN/GaN HEMTs under high-frequency and high-power operations.

## 1. Introduction

Wide bandgap semiconductors are increasingly employed in RF and power electronics industries due to their high breakdown voltage, fast electron saturation velocity, and strong thermal stability [1,2]. GaN-based high-electron-mobility transistors (HEMTs) are particularly valued for their exceptional frequency response [3]. In AlGaN/GaN heterostructures, polarization effects generate a two-dimensional electron gas (2DEG), which serves as a high-mobility channel enabling superior RF performance [4].

These properties have motivated extensive research to improve the RF performance of AlGaN/GaN HEMTs. One reported strategy reduces gate capacitance by asymmetrically removing the passivation layer [5], but this leaves donor-like traps on the AlGaN surface. Such traps from a virtual gate that depletes the 2DEG, causing current collapse [6]. To minimize passivation removal, another method incorporates an under-gate air gap within the T-gate structure to lower capacitance [6,7,8]. However, the low thermal conductivity of air hinders heat dissipation [9], raising device temperatures and compromising long-term reliability. Despite this drawback, studies on the thermal effects introduced by the air gap remain limited.

This study investigates the effect of an air gap gate combined with AlN passivation using technology computer-aided design (TCAD) simulations calibrated with fabricated device data. Unlike finite element analysis (FEA) or purely experimental methods, TCAD provides high-fidelity insight into coupled electrical and thermal behavior, enabling more accurate characterization of thermal properties that are difficult to measure directly [10,11]. The analysis examines heat distribution, extracts thermal resistance, and evaluates operating temperatures under various conditions. To confirm that the intended improvement in RF performance is achieved, the cutoff frequency and DC characteristics are also assessed.

The remainder of this paper is structured as follows. Section 2 outlines the fabrication process and epitaxial structure of the reference AlGaN/GaN HEMT, along with the baseline model calibrated to experimental data. Section 3 presents the electrical and thermal characteristics of devices with an air gap gate and with an air gap gate incorporating AlN passivation. Section 4 discusses the results and concludes with the implications of AlN passivation for thermal management and overall device performance.

## 2. Materials and Methods

### 2.1. Device Fabrication and Modeling

A fabricated 0.15 μm planar-gate AlGaN/GaN HEMT with Si_3_N_4_ passivation was used as the basis for simulation modeling. The unit gate width was set to 100 μm.

**Figure 1:**
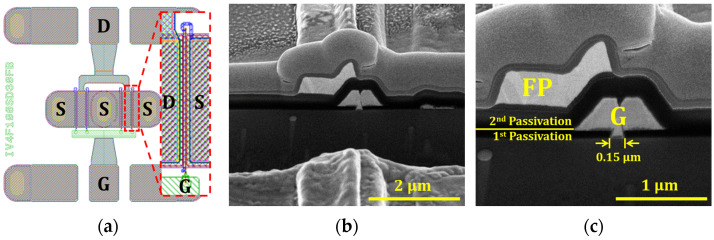
Structural details of the fabricated 0.15 µm planar-gate AlGaN/GaN HEMT: (**a**) Top-view layout showing source (S), gate (G), and drain (D) contacts; (**b**) Focused ion beam-scanning electron microscopy image of the device; (**c**) Enlarged view highlighting the gate, field plate (FP), and passivation layers.

Figure 1 presents the structure of the fabricated 0.15 μm planar-gate AlGaN/GaN HEMT with Si_3_N_4_ passivation. Figure 1a illustrates the source (S), gate (G), and drain (D) contacts, with the modeled region marked by a red dashed rectangle. This region identifies the active area spanning from the drain to the source, encompassing the critical zone where the primary device characteristics are determined. The cross-sectional details obtained via focused ion beam-scanning electron microscopy are provided in Figure 1b. Furthermore, Figure 1c provides a magnified view of the gate, field plate (FP), and passivation layers to clearly visualize the specific locations where structural modifications and material variations were incorporated.

The device incorporates a 0.2 μm nucleation layer, a 2 μm Fe-doped GaN buffer, and a 25 nm Al_0.255_Ga_0.745_N barrier, all grown by metal–organic chemical vapor deposition. Source and drain contacts are fabricated by e-beam evaporation of Ti (30 nm), Al (100 nm), Ni (30 nm), and Au (100 nm), followed by rapid thermal annealing (RTA) at 775 °C for 30 s. Device isolation is implemented through p+ ion implantation using a Si_3_N_4_ layer deposited by plasma-enhanced chemical vapor deposition (PECVD) and photoresist as implantation masks. Thin-film NiCr resistors providing a sheet resistance of 20 Ω/sq are formed using image reversal lithography and lift-off. The first metal interconnect is patterned after via etching through the PECVD Si_3_N_4_ by inductively coupled plasma (ICP). The planar gate is defined via e-beam lithography with a co-polymer/polymethyl methacrylate (PMMA) bilayer resist, followed by Ni/Au deposition and lift-off. The Si_3_N_4_ dielectric layer is then deposited by PECVD, and the source-connected field plate is completed with Ti/Au deposition and lift-off [5].

**Figure 2:**
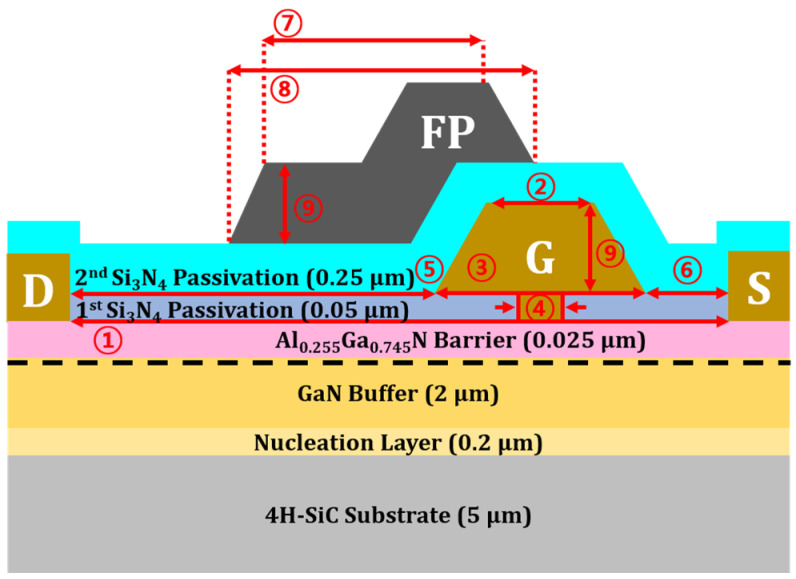
Cross-sectional schematic of the simulated 0.15 μm planar-gate AIGaN/GaN HEMT with Si_3_N_4_ passivation.

Figure 2 illustrates the cross-sectional schematic of the simulated device, with geometrical parameters summarized in Table 1. The model represents a unit cell corresponding to a single finger in the fabricated device HEMT and incorporates both structural and material properties. GaN typically exhibits unintentional n-type doping from native defects such as nitrogen vacancies and silicon impurities [12]. To account for background doping compensation, the GaN buffer in the simulation was Fe-doped at a concentration of 10^19^ cm^−3^ [13,14]. The source and drain are modeled as ohmic contacts, while the gate is defined as a Schottky junction through its work function. To enhance simulation convergence, the active region is represented with a 5 μm substrate instead of the full thickness, following prior TCAD studies [15]. Acceptor-like traps in the Fe-doped GaN buffer suppress vertical electron transport, resulting in negligible current flow below the 2DEG channel. Therefore, restricting the model to the active region with reduced substrate thickness provides sufficient accuracy for device analysis.

**Figure 3:**
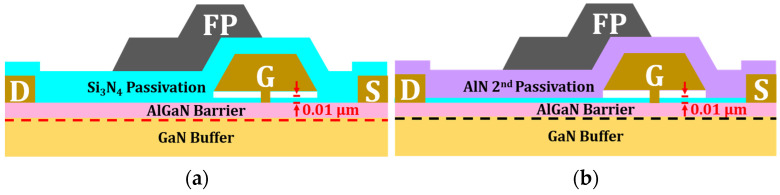
Cross-sectional schematics of simulation structures: (**a**) air gap gate; (**b**) air gap gate with AlN passivation.

**Figure 4:**
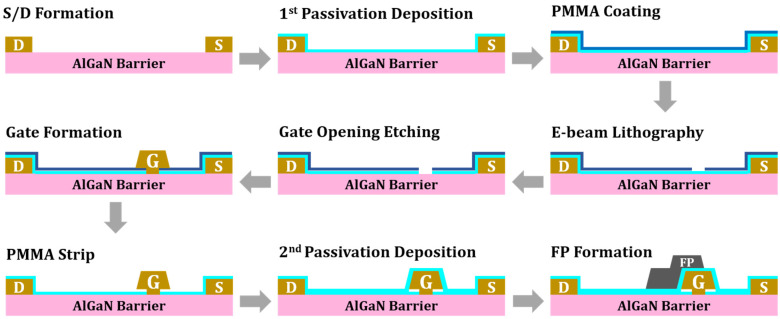
Main fabrication steps for the air gap gate.

Based on the reference structure, additional models incorporating an air gap gate and an air gap gate with AlN passivation were developed for comparison. Figure 3a,b depict the cross-section of these structures, and Figure 4 outlines the main fabrication steps [16,17]. The process begins with an AlGaN barrier layer containing the source and drain contacts. A 10 nm Si_3_N_4_ passivation layer is first deposited to suppress surface traps, followed by a PMMA coating. The gate region is defined through e-beam lithography and etching, after which a T-shaped metal gate is deposited. Removing the PMMA sacrificial layer creates the air gap beneath the gate head. Finally, a second passivation layer of AlN or Si_3_N_4_ is deposited, and the field plate is formed using a lift-off process. The benefits from the stable interface properties of Si_3_N_4_ while leveraging the superior thermal conductivity of AlN, which aligns with established passivation engineering techniques for optimizing interface quality and RF stability [18]. Furthermore, AlN passivation has been experimentally shown to reduce channel temperatures, and its high-quality morphology is also expected to mitigate dynamic issues like current collapse and switching degradation [19,20].

### 2.2. Simulation Setup

The simulation incorporates electrical models and parameters to validate device operation, along with physical models such as the Fermi–Dirac distribution, Shockley–Read-Hall (SRH) recombination, polarization effects, and tunneling mechanisms to accurately capture device behavior. Table 2 summarizes the models and parameters used in the simulation.

Both electrical and thermal behaviors are critical for evaluating AlGaN/GaN HEMTs [21]. To account for thermal effects, a lattice heat flow model and a heat generation model were applied. The lattice heat flow is expressed as:

(1) $$C\frac{\partial T_{L}}{\partial t}=\nabla \left(\kappa \nabla T_{L}\right)+H$$

where C represents the volumetric heat capacitance, κ denotes the thermal conductivity, $T_{L}$ signifies the lattice temperature, and H refers to the heat generation term. Under the drift-diffusion approximation, the heat generation term reduces to:

(2) $$H=(J_{n}+J_{p})\cdot E$$

where $J_{n}$ and $J_{p}$ denote the electron and hole current densities, respectively, and E represents the electric field. Since the lattice heat flow depends on thermal conductivity, it must be considered in the simulation. To capture its temperature dependence, a power-law thermal conductivity model is applied, expressed as:

(3) $$\kappa \left(T_{L}\right)=\frac{TC.CONST}{{\left(\frac{T_{L}}{300}\right)}^{TC.NPOW}}$$

where TC.CONST represents the thermal conductivity of each material at 300 K, and TC.NPOW denotes the calibration factor as temperature dependent, with values determined experimentally. The power-law thermal conductivity model is employed to accurately account for the temperature-dependent reduction in thermal conductivity of semiconductor materials. In wide-bandgap semiconductors like GaN, heat transport is dominated by lattice vibrations. As the lattice temperature increases, phonon scattering intensifies, leading to a degradation in thermal conductivity. This approach is widely adopted in the thermal analysis of GaN HEMTs to capture self-heating effects [22,23]. Table 3 summarizes the power-law thermal conductivity parameters used for each material in the simulation [24]. The AlN passivation layer in this study was modeled as polycrystalline, reflecting the typical characteristics of AlN deposited during AlGaN/GaN HEMT fabrication. AlN provides robust thermal stability and adhesion, ensuring high interfacial compatibility with the underlying Si_3_N_4_ layer. Thermal conductivity of AlN was set to 2.85 W/cm·K, a value representative of high-quality polycrystalline AlN as established in the literature [25].

Appropriate thermal boundary conditions must be defined to model heat dissipation from the device to the surroundings. In this study, the specified boundary conditions are:

(4) $$\left(J_{tot}^{\vec{u}}\cdot \vec{S}\right)=\frac{1}{TBR}(T_{L}-TEMPER)$$

where $J_{tot}^{\vec{u}}$ represents the total energy flux, $\vec{S}$ denotes the unit external normal vector at the boundary, and thermal boundary resistance (TBR) signifies the heat transfer resistance across the material interfaces. The external boundary temperature is denoted by TEMPER [24]. In the simulation, the ambient temperature is set to 300 K, with heat dissipation occurring through both the top surface and the substrate bottom. The reported TBR values for 4H-SiC substrates with the nucleation in AlGaN/GaN HEMTs vary depending on the characterization method: ~5.3 m^2^K/GW via TDTR (Cho et al. [26]), 10–50 m^2^K/GW via Raman spectroscopy (Manoi et al. [27]), and ~4.3 m^2^K/GW via FDTR (Ziade et al. [28]). Additionally, Mu et al. [29] used the TDTR method to report values ranging from ~4.3 to ~5.9 m^2^K/GW. Accordingly, the TBR at the 4H-SiC interface was set to a fitted value of ~7.7 m^2^K/GW, which lies within the reported range. Specifically, the accuracy of the thermal simulation was supported by referencing previous studies that analyzed the thermal characteristics of GaN-based power devices using similar simulation approaches [30,31]. This electro-thermal simulation was further calibrated with experimental data to ensure accuracy.

**Figure 5:**
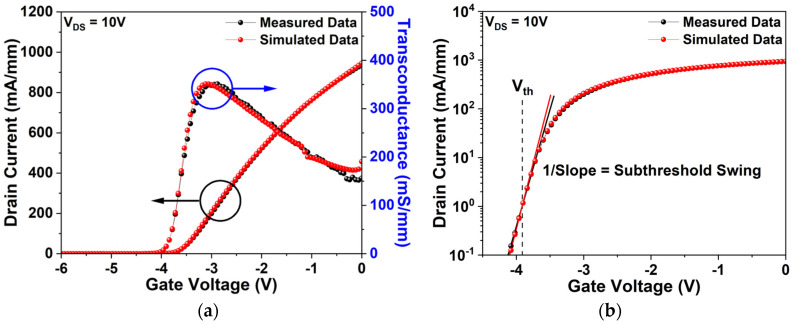
Simulation and experimental I_DS_–V_GS_ transfer characteristics at V_DS_ = 10 V considering self-heating: (**a**) linear scale; (**b**) logarithmic scale.

Figure 5a illustrates the I_DS_–V_GS_ characteristics at V_DS_ = 10 V on a linear scale, with V_GS_ swept from −6 V to 0 V. To enhance resolution near the threshold region, the curve is magnified in Figure 5b, which presents the same data in logarithmic scale. Experimental results yield a saturation drain current (I_dss_) of 937.05 mA/mm, maximum transconductance (G_m_) of 350.71 mS/mm, and threshold voltage (Vth) of −3.92 V. In comparison, simulation predicts I_dss_ of 945.16 mA/mm, G_m_ of 350.85 mS/mm, and Vth of −3.92 V. The corresponding error rates are 0.87%, 0.04%, and 0%, respectively. Subthreshold swing values also exhibit close agreement, measured at 197.09 mV/dec and simulated at 196.01 mV/dec, with deviation within 1%.

**Figure 6:**
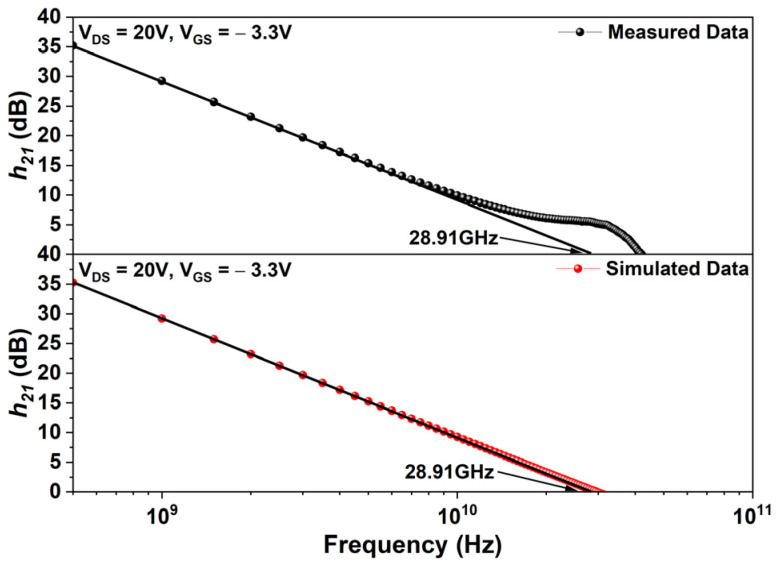
Comparison of current gain from simulation and measurement at V_DS_ = 20 V, V_GS_ = −3.3 V.

Figure 6 compares the current gain ($h_{21}$) obtained from simulation and measurement. RF performance was evaluated through AC small-signal analysis, where capacitances and conductances between electrode pairs under the applied signal were extracted. Using these values along with device width, complex Y-parameters were determined and subsequently converted into S-parameters according to the following relations:

(5) $${∆}_{1}=\frac{1}{\left(1+Y_{11}\right)\left(1+Y_{22}\right)-Y_{12}Y_{21}}$$

(6) $$S_{11}=[\left(1-Y_{11}\right)\left(1+Y_{22}\right)+Y_{12}Y_{21}]{∆}_{1}$$

(7) $$S_{12}=2Y_{12}{∆}_{1}$$

(8) $$S_{21}=2Y_{21}{∆}_{1}$$

(9) $$S_{21}=[\left(1+Y_{11}\right)\left(1-Y_{22}\right)+Y_{12}Y_{21}]{∆}_{1}$$

From the extracted S-parameters, $h_{21}$ was calculated as follows:

(10) $${∆}_{6}=\frac{-2{\left|S_{21}\right|}^{2}}{\left(1-S_{11}\right)\left(1+S_{22}\right)+S_{12}S_{21}}$$

(11) $$h_{21}=20\log_{10}\left(\left|{∆}_{6}\right|\right)$$

Simulated and measured data were obtained at V_DS_ = 20 V and V_GS_ = −3.3 V, corresponding to maximum transconductance. The cutoff frequency ($f_{T}$) was extracted over 1 Hz–50 GHz to generate the $h_{21}$ curve. A linear fit in the 1–10 GHz range was extrapolated, and the *x*-axis intercept at a slope of −20 dB/decade was defined $f_{T}$. Both simulation and measurement yielded $f_{T}=28.91\;GHz$, indicating excellent agreement. With simulation errors confined to 0–0.87% in DC and RF metrics, the model was validated for further analysis.

## 3. Results

Using the 0.15 µm planar-gate HEMT with Si_3_N_4_ passivation as the baseline, the impact of the air gap gate was evaluated. The L_Gate_Foot_ of 0.15 µm is considered optimal for RF performance analysis, as it enhances the cutoff frequency by shortening the electron transit path while mitigating the strong short-channel effects that arise when the gate length falls below 0.1 µm [32]. To confirm its effectiveness in reducing gate capacitance, the gate-to-source capacitance ($C_{gs}$) was simulated at V_DS_ = 20 V and V_GS_ = −3.3 V.

**Figure 7:**
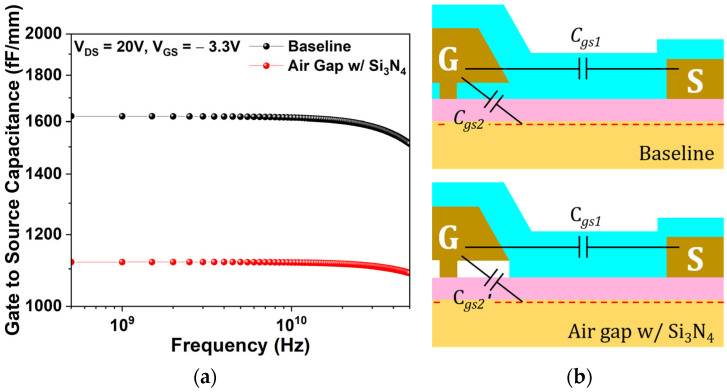
(**a**) $C_{gs}$ versus frequency at V_DS_ = 20 V, V_GS_ = −3.3 V for the baseline and the air gap gate; (**b**) schematic of $C_{gs}$ components for both structures.

Figure 7a depicts the simulated $C_{gs}$ as a function of frequency, while Figure 7b illustrates its constituent components. Introducing the air gap gate reduces $C_{gs}$ by approximately 30% compared to the baseline device. As depicted in Figure 7b, C_gs1_ represents the interelectrode capacitance between the source and gate, whereas C_gs2_ arises from depletion beneath the gate and reflects coupling between the gate and source through the 2DEG [33]. With the air gap gate, C_gs1_ remains constant, while C_gs2_ decreases (denoted as C_gs2_′) due to the replacement of part of the Si_3_N_4_ passivation with lower permittivity air. This reduction in $C_{gs}$ leads to the overall decrease in $f_{T}$ as follows:

(12) $$f_{T}=\frac{g_{m}}{2\pi (C_{gs}+C_{gd})}\approx \frac{g_{m}}{2\pi C_{gs}}$$

where $g_{m}$ denotes the transconductance, and $C_{gd}$ represents the gate-to-drain capacitance, which can be neglected.

**Figure 8:**
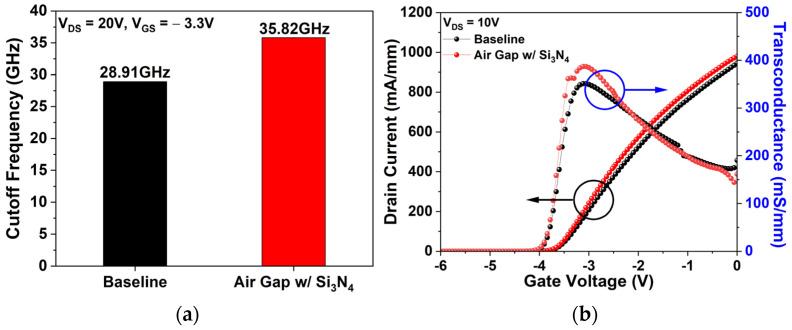
(**a**) $f_{T}$ for the baseline and air gap gate at V_DS_ = 20 V, V_GS_ = −3.3 V; (**b**) I_DS_–V_GS_ transfer characteristics of the baseline and air gap gate at V_DS_ = 10 V.

Figure 8a presents $f_{T}$ for the baseline and air gap gate under identical bias conditions (V_DS_ = 20 V, V_GS_ = −3.3 V). The baseline records 28.91 GHz, while the air gap gate attains 35.82 GHz, corresponding to a 23.9% enhancement. Since $f_{T}$ is governed by both $C_{gs}$ and $g_{m}$, the influence of the air gap gate on I_DS_–V_GS_ transfer characteristics was also analyzed.

Figure 8b compares the I_DS_–V_GS_ transfer characteristics of the baseline and air gap gate at V_DS_ = 10 V. The air gap gate causes a minor Vth shift from −3.92 V to −3.94 V, while I_dss_ and G_m_ increase substantially to 980.09 mA/mm (3.7%) and 386.88 mS/mm (10.27%), respectively, from 945.16 mA/mm and 350.85 mS/mm.

Table 4 summarizes the 2DEG concentration for the baseline and air gap gate. The air gap gate shows a 13.56% increase, reaching 3.2 × 10^12^ cm^−2^. The higher 2DEG density enhances current transport, raising I_dss,_ while also improving G_m_ by enabling gate voltage variations to more effectively modulate the drain current [34].

**Figure 9:**
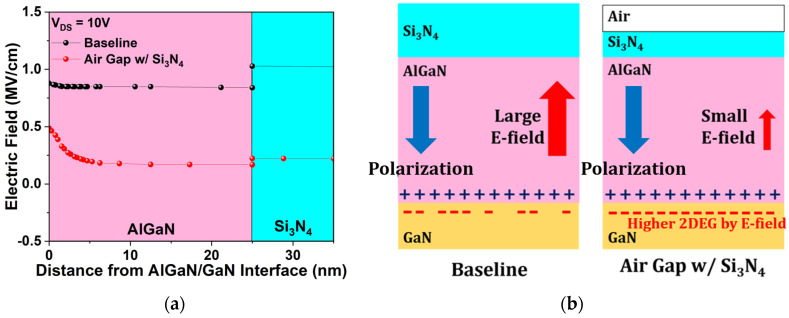
(**a**) Electric field distribution in the AlGaN/GaN heterostructure for the baseline and the air gap gate; (**b**) Schematic illustrating electric field redistribution and its effect on 2DEG concentration at the AlGaN/GaN interface due to the air gap gate.

The change in 2DEG concentration arises from the permittivity variation introduced by the modified passivation scheme. Figure 9a compares the electric field in the AlGaN/GaN heterostructure for the baseline and air gap gate, while Figure 9b schematically depicts its influence on 2DEG concentration at the AlGaN/GaN interface. In the air gap gate, replacement of part of the Si_3_N_4_ passivation with air redistributes the electric field due to the permittivity contrast between the two materials [35]. Part of the electric field is distributed into the lower-permittivity air (Figure 9a), reducing the field across the AlGaN barrier relative to the baseline. Intrinsic polarization charges remain constant as they are determined by spontaneous and piezoelectric polarization. However, this modified vertical electric field distribution less effectively counteracts the polarization-induced internal field [36], allowing for a higher 2DEG accumulation at the AlGaN/GaN interface compared to the baseline. Consequently, the air gap gate structure supports a higher 2DEG concentration [37].

The air gap gate alters both electrical and thermal behavior. Since air has low thermal conductivity, it impedes channel heat dissipation and introduces thermal challenges. To mitigate these effects, the air gap gate is combined with a high-conductivity AlN passivation layer.

**Figure 10:**
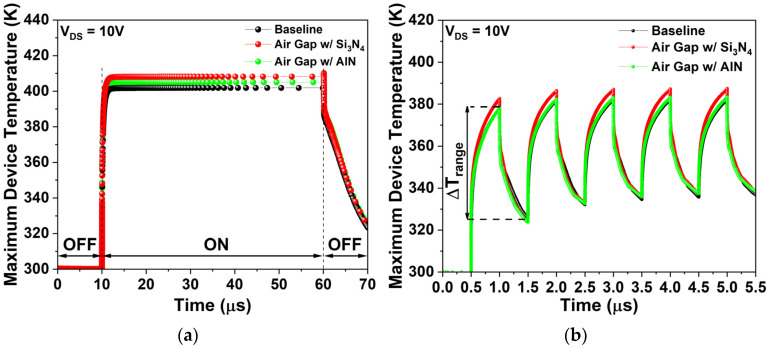
(**a**) Device temperature under on-state operation (V_GS_ = 0 V) for 50 µs at V_DS_ = 10 V; (**b**) Device temperature during five on/off cycles (V_GS_ = 0 V/−5 V) with a 1 µs period at V_DS_ = 10 V.

Figure 10a represents the simulated device temperature in the on-state (V_GS_ = 0 V) for 50 µs at V_DS_ = 10 V, while Figure 10b shows simulated temperature variation over five on-off cycles (V_GS_ = 0 V and V_GS_ = −5 V) with a 1 µs period at V_DS_ = 10 V. Table 5 summarizes the average on-state operating temperature (T_ON_) for 50 µs from Figure 10a and the average temperature difference (ΔT_range_) between maximum and minimum values over the five cycles from Figure 10b.

During prolonged on-state operation (Figure 10a), the air gap gate shows a T_ON_ 3.3% (12.19 K) higher than the baseline. Under repetitive cycling (Figure 10b), its ΔT_range_ averaged over five cycles is 8.81% (4.25 K) greater. Elevated T_ON_ degrades performance, while larger ΔT_range_ shortens device lifetime and stability. Incorporating AlN passivation alleviates this thermal degradation. With AlN, the air gap gate records a T_ON_ only 0.6% (2.22 K) above the baseline, representing a 2.62% (9.97 K) improvement over the air gap with Si_3_N_4_ passivation. Likewise, average ΔT_range_ rises by only 0.44% (0.21 K) relative to the baseline, improving by 7.69% (4.03 K) compared with the air gap gate.

As shown in Figure 10a, the maximum device temperature rises rapidly after turn-on and saturates within ~5 µs, indicating that thermal generation and dissipation have reached thermal equilibrium. This observation is consistent with previous studies reporting that device heating begins within ~70 ns after turn-on and that the transient temperature evolution in GaN-on-SiC HEMTs is completed within ~10 µs [38,39]. Therefore, a 50 µs observation is sufficiently long to capture the transient response and the saturated T_ON_.

**Figure 11:**
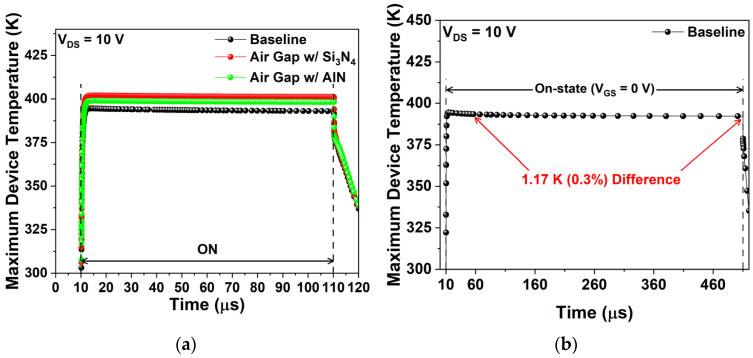
(**a**) Device temperature under on-state operation (V_GS_ = 0 V) for 100 µs at V_DS_ = 10 V; (**b**) Device temperature of baseline under on-state operation (V_GS_ = 0 V) for 500 μs at V_DS_ = 10 V.

Figure 11a represents the simulated device temperature of three structures in the on-state (V_GS_ = 0 V) for 100 µs at V_DS_ = 10 V, while Figure 11b shows the simulated device temperature of the baseline in the on-state (V_GS_ = 0 V) for 500 µs at V_DS_ = 10 V. Table 6 summarizes the average T_ON_ for 100 µs from Figure 11a. As the on-state duration increases, the initial heating stage (~5 µs after turn-on) becomes relatively shorter. Consequently, the average T_ON_ converges to the saturated T_ON_. With AlN, the air gap gate records average T_ON_ 1.78% (6.88 K) above the baseline, representing a 1.45% (5.76 K) improvement over the air gap with Si_3_N_4_ passivation for 100 µs. The thermal improvement is more pronounced during short switching, which is acceptable considering the typical short on-times of GaN HEMTs [40]. Furthermore, as indicated in Figure 11b, when the on-state was extended tenfold to 500 µs, the T_ON_ differed by only 1.17 K (0.30%) after 50 µs for baseline.

Nonetheless, the air gap gate also modified electrical behavior, necessitating evaluation of whether these differences arise from improved thermal management or increased Joule heating due to higher drain current. Thermal resistance ($R_{Th}$) values derived from the transient thermal response range from 10.89 to 11.24 K·mm/W for all three structures, falling within the reported range for measured devices (9 to 14 K·mm/W) [41,42,43,44].

**Figure 12:**
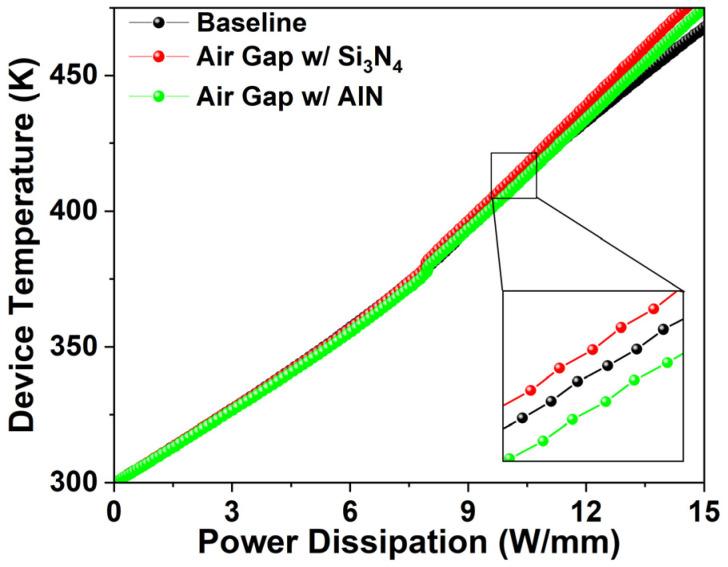
Device temperature versus power dissipation.

Figure 12 presents the device temperature under power dissipation. The air gap gate shows higher temperatures than the baseline under identical power, with increases of 1.94 K (0.48%) at 10 W/mm, and 13.59 K (2.91%) at 15 W/mm, indicating worsening thermal degradation at higher power densities. AlN passivation mitigates this effect: relative to the baseline, the air gap gate with AlN shows a 1.99 K (0.49%) decrease at 10 W/mm and a 6.29 K (1.56%) increase at 15 W/mm. Compared with an air gap gate with Si_3_N_4_ passivation, AlN improves heat dissipation by 3.93 K (0.96%) at 10 W/mm and by 7.3 K (1.31%) at 15 W/mm, demonstrating greater benefit at higher power densities.

(13) $$R_{Th}=\frac{\Delta T}{P_{D}}$$

where ΔT represents the device temperature rise, and $P_{D}$ denotes the power dissipation.

**Figure 13:**
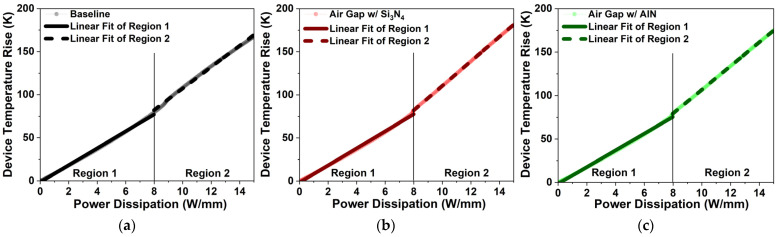
Linear segments of ΔT versus $P_{D}$ for extracting $R_{Th}$: (**a**) Baseline; (**b**) air gap gate; (**c**) air gap gate with AlN passivation.

Using the linear portion of the ΔT versus $P_{D}$ plot in Figure 13a–c, the $R_{Th}$ of the three structures was calculated. Two distinct linear regions were used to extract $R_{Th}$ values, which are summarized in Table 7. All extracted values fall within the experimentally reported range for GaN-on-SiC HEMTs (9–14 K·mm/W), confirming the reliability of the simulation results [41,42,43,44].

The air gap gate increases $R_{Th}$ by 0.07 K·mm/W (0.71%) in Region 1 and 1.81 K·mm/W (14.57%) in Region 2 relative to the baseline. The difference between regions (${\Delta R}_{Th}$) rises by 1.74 K·mm/W (68.24%), indicating degraded heat dissipation and reduced thermal stability. Incorporating AlN passivation mitigates this degradation, yielding slightly lower thermal resistance of 0.19 K·mm/W (1.93%) in Region 1 and a smaller increase of 1.25 K·mm/W (10.06%) in Region 2 compared to the baseline. This represents improvements of 0.26 K·mm/W (2.62%) in Region 1 and 0.56 K·mm/W (3.94%) in Region 2 relative to the air gap gate with Si_3_N_4_ passivation. In addition, ΔR_Th_ is also reduced by 0.3 K·mm/W (6.99%), reflecting enhanced thermal stability.

**Figure 14:**
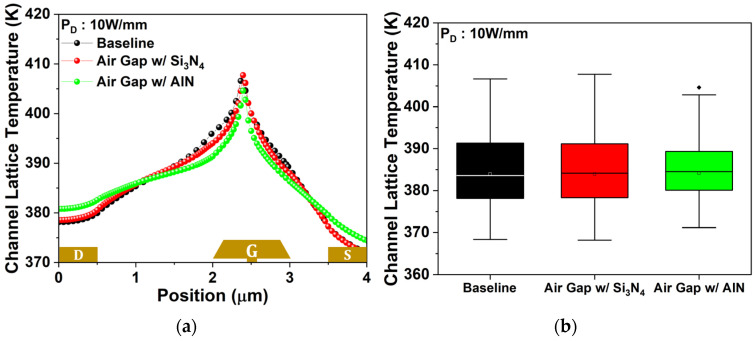
(**a**) Lattice temperature profile of the 2DEG channel at a $P_{D}$ of 10 W/mm; (**b**) corresponding box plot. Small diamond is an outlier.

Finally, temperature distribution within the device, which strongly affects the device lifetime, was analyzed [45]. Figure 14a presents lattice temperature in the 2DEG channel at 10 W/mm, and Figure 14b provides the corresponding boxplot. The local temperature difference between the hottest and coolest points in the 2DEG channel was 38.25 K for the baseline, 39.58 K for the air gap gate, and 33.47 K for the air gap gate with AlN passivation. The air gap gate exhibited 1.33 K (3.47%) greater localized heating than the baseline. In contrast, the high thermal conductivity of AlN passivation enabled the passivated air gap gate to distribute heat more uniformly, reducing the temperature difference by 4.78 K (12.5%) relative to the baseline and by 6.11 K (15.44%) compared to the air gap gate with Si_3_N_4_ passivation. These results confirm that AlN passivation effectively mitigates the thermal limitations of the air gap gate. To ensure that the enhancement of RF performance is preserved, the $C_{gs}$ and $f_{T}$ of all three structures were also analyzed.

**Figure 15:**
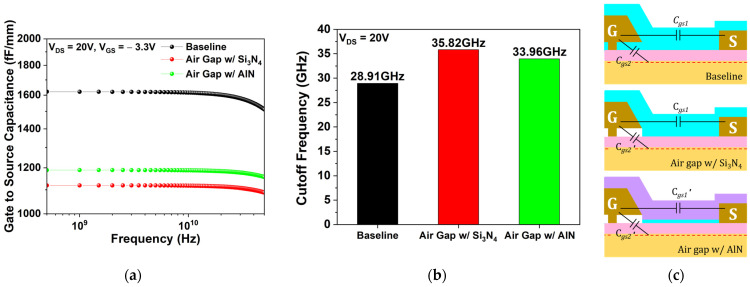
(**a**) $C_{gs}$ as a function of frequency at V_DS_ = 20 V and V_GS_ = −3.3 V; (**b**) Schematic of $C_{gs}$ components for the baseline, air gap gate, and the air gap gate with AlN passivation (from top to bottom); (**c**) $f_{T}$ of the three structures at V_DS_ = 20 V and V_GS_ = −3.3 V.

Figure 15a shows $C_{gs}$ at V_DS_ = 20 V and V_GS_ = −3.3 V, while Figure 15b illustrates its constituent components. Figure 15c presents the corresponding $f_{T}$ of the three structures under the same conditions. The air gap gate with AIN passivation exhibits an $f_{T}$ of 33.96 GHz, 1.86 GHz (5.19%) lower than the air gap gate with Si_3_N_4_ passivation. This reduction (Figure 15b,c) results from an increase in interelectrode capacitance (C_gs1_) between the gate and source, caused by the higher permittivity of AlN (denoted as C_gs1_′). However, the influence of C_gs1_ remains minor relative to C_gs2_ [33]. Consequently, the total $C_{gs}$ for the AIN-passivated air gap gate remains ~25.61% lower than the baseline, yielding an $f_{T}$ 5.05 GHz (17.47%) higher than the baseline and demonstrating a significant enhancement in RF performance.

**Figure 16:**
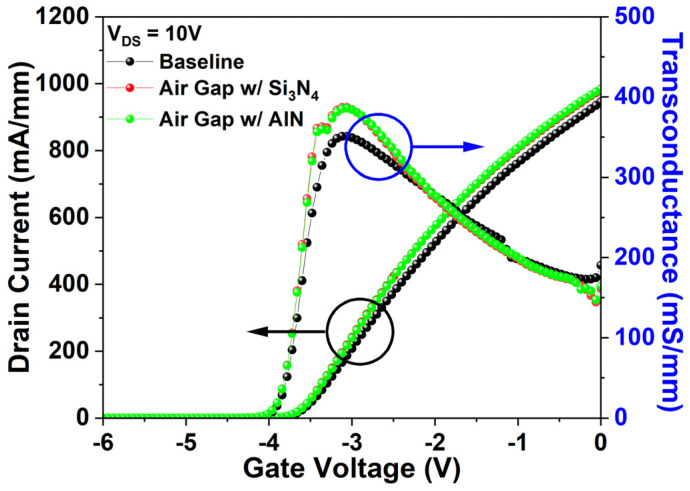
I_DS_–V_GS_ transfer characteristics of three structures at V_DS_ = 10 V.

The impact of AlN passivation on DC characteristics was also evaluated. Figure 16 compares the I_DS_–V_GS_ transfer characteristics of the air gap gate with AlN passivation at V_DS_ = 10 V. The Vth remained at −3.94 V, while I_dss_ increased slightly to 983.72 mA/mm and G_m_ decreased marginally to 385.85 mS/mm. These changes of 0.27% and 0.37%, respectively, are negligible, indicating that AlN passivation does not significantly affect the DC performance of the air gap gate.

## 4. Discussion

This study examined the influence of the air gap gate on AlGaN/GaN HEMTs. The results demonstrate that the air gap gate substantially improves electrical performance, yielding a 23.9% increase in $f_{T}$ along with higher I_dss_ and G_m_. These outcomes align with prior findings that lowering the dielectric permittivity between the gate and channel reduces $C_{gs}$ and enhances $f_{T}$. In contrast to earlier studies, the present work provides a detailed quantification of thermal degradation. Electro-thermal TCAD simulations reveal that the air gap gate elevates operating temperature, thermal resistance, and temperature gradients, which may compromise long-term reliability.

This study further showed that integrating high-thermal-conductivity AlN passivation with the air gap gate effectively mitigates thermal limitations. AlN passivation reduced average operating temperature, suppressed temperature variation, lowered thermal resistance, and alleviated localized heating in the 2DEG channel. Importantly, these thermal benefits were achieved without compromising the RF enhancement, as $f_{T}$ remained 17.5% above the baseline.

In conclusion, integrating AlN passivation with the air gap gate mitigates the thermal drawbacks of the conventional structure while retaining the RF performance benefits of the reduced capacitance. This combined approach offers a promising pathway toward high-reliability, high-performance AlGaN/GaN HEMTs for advanced RF and high-power applications.

## Figures and Tables

**Table 1:** Geometrical parameters of the simulated device structure.

Parameters		Values (μm)	Parameters		Values (μm)	Parameters		Values (μm)
①	L_Source-Drain_	3.0	④	L_Gate_Foot_	0.15	⑦	L_Field_Plate_Top_	1.4
②	L_Gate_Head_Top_	0.8	⑤	L_Gate-Drain_	1.5	⑧	L_Field_Plate_Bottom_	1.6
③	L_Gate_Head_Bottom_	1.0	⑥	L_Source-Gate_	0.5	⑨	H_Field_Plate_	1.5

**Table 2:** Simulation models and parameters.

Parameters	Units	GaN	AlGaN
Bandgap energy	eV	3.44	4
Electron Affinity	eV	3.05	2.66
Relative permittivity	-	8.9	8.8
Electron SRH lifetime	s	1.2 × 10^−8^	1.2 × 10^−8^
Hole SRH lifetime	s	1.2 × 10^−8^	1.2 × 10^−8^
Electron saturation velocity	cm/s	1.91 × 10^7^	1.12 × 10^7^
Hole saturation velocity	cm/s	1 × 10^6^	1 × 10^6^
Low field mobility model	-	Farahmand Modified Caughey Thomas model	
High field mobility model	-	GANSAT model	
Electron relative effective mass	-	0.2	0.229
Hole relative effective mass	-	1	0.851
Lattice constant	Å	3.19	3.17

**Table 3:** Thermal conductivity model parameters.

Parameters	Units	4H-SiC	GaN	AlGaN	Si_3_N_4_	AlN	Air
TC.CONST	W/cm∙K	2.6	1.3	0.4	0.3	2.85	2.64 × 10^−4^
TC.NPOW	-	0	0.28	0	0	1.64	−0.83

**Table 4:** 2DEG concentration of the baseline and the air gap gate.

	2DEG Density (cm^−2^)
Baseline	2.36 × 10^13^
Air gap gate	2.68 × 10^13^

**Table 5:** Operational device temperature characteristics.

	Average T_ON_ (K)	Average ΔT_range_ (K)
Baseline	368.87	48.20
Air gap gate with Si_3_N_4_ passivation	381.06	52.45
Air gap gate with AlN passivation	371.09	48.42

**Table 6:** Operational device temperature characteristics under on-state operation for 100 µs.

	Average T_ON_ (K)
Baseline	385.74
Air gap gate with Si_3_N_4_ passivation	398.38
Air gap gate with AlN passivation	392.62

**Table 7:** Summary of $R_{Th}$ and ${\Delta R}_{Th}$ of three structures of HEMT.

	R_Th_ (K∙mm/W)		ΔR_Th_
	Region 1	Region 2	
Baseline	9.87	12.42	2.55
Air gap gate	9.94	14.23	4.29
Air gap gate with AlN passivation	9.68	13.67	3.99

## Data Availability

The original contributions presented in this study are included in the article. Further inquiries can be directed to the corresponding author.

## References

[1] Zhou J., Zhong L., Feng X., Zhang W., Liu X., Zhou H., Liu Z., Hao Y., Zhang J. (2025). Recent Advances in Device-Level Thermal Management Technologies for Wide Bandgap Semiconductor: A Review. IEEE Trans. Electron Devices.

[2] Buffolo M., Favero D., Marcuzzi A., De Santi C., Meneghesso G., Zanoni E., Meneghini M. (2024). Review and outlook on GaN and SiC power devices: Industrial state-of-the-art, applications, and perspectives. IEEE Trans. Electron Devices.

[3] Kozak J.P., Zhang R., Porter M., Song Q., Liu J., Wang B., Wang R., Saito W., Zhang Y. (2023). Stability, reliability, and robustness of GaN power devices: A review. IEEE Trans. Power Electron..

[4] Jones E.A. (2016). Review of commercial GaN power devices and GaN-based converter design challenges. IEEE J. Emerg. Sel. Top. Power Electron..

[5] Choi J.-H., Kim D., Lee S.-J., Kim J.-H., Cho Y.-A., Min B.-G., Kang D.M., Kim H.-S. (2023). Improved RF performances by applying asymmetric passivation and air-bridged field plate in AlGaN/GaN HEMTs with reliability-based simulation. IEEE Trans. Electron Devices.

[6] Shakya A., Pal P., Kabra S. (2024). Comparative Analysis of T-gate GaN HEMT with different passivation layers for RF Applications. Proceedings of the 2024 International Conference on Communication, Control, and Intelligent Systems (CCIS), Mathura, India, 6–7 December 2024.

[7] Huang S., Wei K., Liu G., Zheng Y., Wang X., Pang L., Kong X., Liu X., Tang Z., Yang S. (2014). High-fMAX High Johnson’s Figure-of-Merit 0.2-um Gate AlGaN/GaN HEMTs on Silicon Substrate with AlN/SiN Passivation. IEEE Electron Device Lett..

[8] Liu X., Chen J., Jiang Y., Bian K., Wang H. (2024). Novel T-Shaped gate with air gap for AlGaN/GaN HEMTs on silicon with high Johnson’s figures of merit. IEEE Trans. Electron Devices.

[9] Mavromatidis L.E., Bykalyuk A., El Mankibi M., Michel P., Santamouris M. (2012). Numerical estimation of air gaps’ influence on the insulating performance of multilayer thermal insulation. Build. Environ..

[10] Chen X., Boumaiza S., Wei L. (2020). Modeling bias dependence of self-heating in GaN HEMTs using two heat sources. IEEE Trans. Electron Devices.

[11] Formicone G., Walker J., Pomeroy J., Kuball M. (2024). On the use of TCAD for the thermal analysis of RF power transistors and its application to a GaN-on-SiC HEMT with a diamond composite flange. Proceedings of the 19th European Microwave Integrated Circuits Conference (EuMIC), Paris, France, 23–24 September 2024.

[12] Zou X., Yang J., Qiao Q., Zou X., Chen J., Shi Y., Ren K. (2023). Trap characterization techniques for GaN-Based HEMTs: A critical review. Micromachines.

[13] Painter V.V., Sommet R., Chang C., Brunel V.D.G., Gaillard F., Nallatamby J.-C., Raja P.V. (2025). Fe-doping starting depth impacts on static and transient characteristics of AlGaN/GaN HEMTs. Micro Nanostruct..

[14] Dai J.-J., Mai T.T., Nallasani U.R., Chang S.-C., Hsiao H.-I., Wu S.-K., Liu C.-W., Wen H.-C., Chou W.-C., Wang C.-P. (2022). The effect of heavy Fe-doping on 3D growth mode and Fe diffusion in GaN for high power HEMT application. Materials.

[15] Powar K.S., Tadepalli V.K., Painter V.V., Sommet R., Chakravorty A., Raja P.V. (2025). Maximum, effective, and average thermal resistance for GaN-based HEMTs on SiC, Si and sapphire substrates. Solid-State Electron..

[16] Sun B., Zhang P., Zhang T., Shangguan S., Wu S., Ma X. (2020). Single step electron-beam lithography archiving lift-off for T-gate in high electron mobility transistor fabrication. Microelectron. Eng..

[17] Xiang S.L., Ding Q.-C., Liu J., Hu H., Zhao B., Lu S.Z., Huang F., Cunha J., Rodrigues J., Yu Z. (2024). Photoresist systems in floating T-gate fabrication for GaN high electron mobility transistors. Nano Mater. Sci..

[18] Wang Q., Cheng X., Zheng L., Ye P., Li M., Shen L., Li J., Zhang D., Gu Z., Yu Y. (2017). Band alignment between PEALD-AlNO and AlGaN/GaN determined by angle-resolved X-ray photoelectron spectroscopy. Appl. Surf. Sci..

[19] Zhang D., Cheng X., Zheng L., Shen L., Wang Q., Gu Z., Qian R., Wu D., Zhou W., Cao D. (2018). Effects of polycrystalline AlN film on the dynamic performance of AlGaN/GaN high electron mobility transistors. Mater. Des..

[20] Tsurumi N., Ueno H., Murata T., Ishida H., Uemoto Y., Ueda T., Inoue K., Tanaka T. (2010). AlN passivation over AlGaN/GaN HFETs for surface heat spreading. IEEE Trans. Electron Devices.

[21] Sadi T. (2006). Investigation of self-heating effects in submicrometer GaN/AlGaN HEMTs using an electrothermal Monte Carlo method. IEEE Trans. Electron Devices.

[22] Bagnall K.R. (2013). Device-Level Thermal Analysis of GaN-Based Electronics. Ph.D. Thesis.

[23] Vitanov S. (2010). Simulation of High Electron Mobility Transistors. Ph.D. Thesis.

[24] Silvaco (2020). Atlas User’s Manual Device Simulation Software.

[25] Cheng Z., Koh Y.R., Mamun A., Shi J., Bai T., Huynh K., Yates L., Liu Z., Li R., Lee E. (2020). Experimental observation of high intrinsic thermal conductivity of AlN. Phys. Rev. M.

[26] Cho J., Li Y., Hoke W.E., Altman D.H., Asheghi M., Goodson K.E. (2014). Phonon Scattering in Strained Transition Layers for GaN Heteroepitaxy. Phys. Rev. B.

[27] Manoi A., Pomeroy J.W., Killat N., Kuball M. (2010). Benchmarking of Thermal Boundary Resistance in AlGaN/GaN HEMTs on SiC Substrates: Implications of the Nucleation Layer Microstructure. IEEE Electron Device Lett..

[28] Ziade E., Yang J., Brummer G., Nothern D., Moustakas T., Schmidt A.J. (2015). Thermal Transport Through GaN-SiC Interfaces from 300 to 600 K. Appl. Phys. Lett..

[29] Mu F., Cheng Z., Shi J., Shin S., Xu B., Shiomi J., Graham S., Suga T. (2019). High Thermal Boundary Conductance across Bonded Heterogeneous GaN-SiC Interfaces. ACS Appl. Mater. Interfaces.

[30] Lim C.-Y., Lee J.H., Kim T.S., Won Y.H., Min B.G., Kang D.M., Kim H.S. (2026). Optimization of central source contact length to mitigate temperature variation and thermal crosstalk in multi-finger AlGaN/GaN HEMTs: Reliability-based simulation. MSSP.

[31] Jang K.-W., Hwang I.-T., Kim H.-J., Lee S.-H., Lim J.-W., Kim H.-S. (2019). Thermal analysis and operational characteristics of an AlGaN/GaN High electron mobility transistor with copper-filled structures: A simulation study. Micromachines.

[32] Jung H.-W., Chang S.-J., Kang D.-M. (2024). Impact of Gate Length Scaling on DC and RF Performance in AlGaN/GaN HEMTs. Proceedings of the 15th International Conference on Information and Communication Technology Convergence (ICTC), Jeju Island, Republic of Korea, 16–18 October 2024.

[33] Yan P., Liang W., Tingting Y., Sihua O., Lei P., Guoguo L., Weijun L., Xinyu L. (2010). Multi-bias capacitance voltage characteristic of AlGaN/GaN HEMT. J. Semicond..

[34] Pérez-Tomás A., Fontserè A., Placidi M., Baron N., Chenot S., Moreno J.C., Cordier Y. (2012). Temperature impact and analytical modeling of the AlGaN/GaN-on-Si saturation drain current and transconductance. Semicond. Sci. Technol..

[35] Kim J.-H., Lim C.Y., Lee J.H., Choi J.H., Min B.G., Kang D.M., Kim H.S. (2024). Operational Characteristics of AlGaN/GaN High-Electron-Mobility Transistors with Various Dielectric Passivation Structures for High-Power and High-Frequency Operations: A Simulation Study. Micromachines.

[36] Kaushik P.K., Singh S.K., Gupta A., Basu A., Chang E.Y. (2021). Impact of surface states and aluminum mole fraction on surface potential and 2DEG in AlGaN/GaN HEMTs. Nanoscale Res. Lett..

[37] Heikman S., Keller S., Wu Y., Speck J.S., DenBaars S.P., Mishra U.K. (2003). Polarization effects in AlGaN/GaN and GaN/AlGaN/GaN heterostructures. J. Appl. Phys..

[38] Riedel G.J., Pomeroy J.W., Hilton K.P., Maclean J.O., Wallis D.J., Uren M.J., Martin T., Kuball M. (2008). Nanosecond timescale thermal dynamics of AlGaN/GaN electronic devices. IEEE Electron Device Lett..

[39] Kuball M., Riedel G.J., Pomeroy J.W., Sarua A., Uren M.J., Martin T., Hilton K.P., Maclean J.O., Wallis D.J. (2007). Time-resolved temperature measurement of AlGaN/GaN electronic devices using micro-Raman spectroscopy. IEEE Electron Device Lett..

[40] De Santi C., Meneghini M., Meneghesso G., Zanoni E. (2018). Review of dynamic effects and reliability of depletion and enhancement GaN HEMTs for power switching applications. IET Power Electron..

[41] Joh J. (2009). Measurement of channel temperature in GaN high-electron mobility transistors. IEEE Trans. Electron Devices.

[42] Bouslama M. (2019). Dynamic performance and characterization of traps using different measurements techniques for the new AlGaN/GaN HEMT of 0.15 µm ultrashort gate length. IEEE Trans. Microw. Theory Tech..

[43] Jakani A. (2021). Comparison of GaN HEMTs thermal results through different measurements methodologies: Validation with 3D simulation. Proceedings of the Thermal Investigations of ICs and Systems (Therminic), Berlin, Germany, 23 September 2021.

[44] Martin-Horcajo S. (2013). Simple and accurate method to estimate channel temperature and thermal resistance in AlGaN/GaN HEMTs. IEEE Trans. Electron Devices.

[45] Manoi A. (2011). Time-dependent thermal crosstalk in multifinger AlGaN/GaN HEMTs and implications on their electrical performance. Solid-State Electron..

